# Mechanism of Action of Acupotomy in Inhibiting Chondrocyte Apoptosis in Rabbits with KOA through the PI3K/Akt Signaling Pathway

**DOI:** 10.1155/2020/4241917

**Published:** 2020-11-10

**Authors:** Xiao-shuang Huang, Kai Geng, Shi-yu Luo, Cun-bin Liu, Kai-ning Yang, Ao Zhai, Yong-hui Yang

**Affiliations:** ^1^Anhui University of Traditional Chinese Medicine, Hefei 230038, China; ^2^The Third Affiliated Hospital of Anhui University of Traditional Chinese Medicine, Hefei 230061, China

## Abstract

**Objective:**

We examined the effects of acupotomy on the PI3K/Akt signaling pathway to elucidate the mechanism of action of acupotomy on articular chondrocyte apoptosis among rabbits with knee osteoarthritis (KOA).

**Methods:**

New Zealand rabbits were randomly assigned to a healthy control group, placebo group, acupotomy group, and drug group, with 10 rabbits in each group. Changes in chondrocytes were examined by hematoxylin and eosin staining, and articular chondrocyte apoptosis was measured by electron microscopy and immunofluorescence. The mRNA and protein expression levels of PI3K and Akt were measured by real-time quantitative PCR and Western blot.

**Results:**

In contrast, less chromatin margination and clear and smooth nuclear envelope boundary were visible in the acupotomy group and drug group. The number of apoptotic chondrocytes in the knee joint of rabbits was significantly higher in the placebo group than that in the acupotomy group and drug group (*P* < 0.05). The acupotomy group had a nonsignificantly lower number of apoptotic chondrocytes than the drug group (*P* > 0.05). Furthermore, the mRNA and protein expression levels of PI3K and Akt were significantly higher in the acupotomy group and drug group than those in the placebo group (*P* < 0.05) and were closer to normal levels in the acupotomy group than those in the drug group (*P* < 0.05). PI3K and Akt expression levels were negatively correlated with chondrocyte apoptosis in the knee joint of rabbits in all groups.

**Conclusion:**

Inhibiting chondrocyte apoptosis in the knee joint of KOA rabbits by upregulating the PI3K/Akt signaling pathway may be a possible mechanism of acupotomy in treating KOA.

## 1. Introduction

Osteoarthritis (OA) is a degenerative joint disease characterized by articular cartilage damage that seriously affects the life quality of patients. Knee osteoarthritis (KOA) is the most common clinical type of OA that causes knee pain and functional limitation in patients [1, 2]. Acupotomy has demonstrated a satisfactory effect in the treatment of KOA in recent years, but studies on the mechanism of acupotomy in KOA have mostly focused on signaling pathways such as FAK-PI3K, Notch, and Wnt/*β*-catenin [3–5], as well as its effects on cartilage extracellular matrix type II collagen (COL-II), aggrecan, and cartilage integrin *β*1. Nevertheless, acupotomy has been reported to promote articular cartilage repair and reduce articular cartilage damage, which provide evidence for the mechanism of action of acupotomy in KOA [6–8].

Excessive articular chondrocyte apoptosis is an important characteristic of KOA pathogenesis and results in articular cartilage damage [9]. However, the mechanisms by which acupotomy regulates chondrocyte apoptosis and associated signaling pathways are currently unclear. Therefore, determining whether acupotomy plays a role in the inhibition of chondrocyte apoptosis and suppression of chondrocyte apoptosis-related signaling pathways is a key to elucidating the mechanism of action of acupotomy in KOA and a topic worthy of further investigation.

Phosphatidylinositol-3-kinase/protein kinase B (PI3K/Akt) is a classical antiapoptotic signaling pathway associated with chondrocyte apoptosis in OA [10–12]. PI3K/Akt signaling pathway can affect the activation of multiple downstream effectors and play a key role in suppressing cell apoptosis and promoting cell proliferation [13]. Here, we investigated the action of acupotomy on the PI3K/Akt signaling pathway in articular chondrocytes of KOA rabbits using various molecular biology techniques and analyzed the correlation between chondrocyte apoptosis and PI3K/Akt expression in order to provide insights into the mechanism of action of acupotomy in KOA.

## 2. Materials and Methods

### 2.1. Experimental Animal and Grouping

Forty healthy, three-month-old male New Zealand white rabbits (2.0–2.5 kg) were provided by the Pizhou Dongfang Breeding Co., Ltd. (animal certificate no.: SCXK (Su) 2014-0005). The rabbits were acclimatized for 3 d with *ad libitum* access to food and water at (22 ± 2)°C, (55 ± 15)% humidity, and 12 h light/12 h dark cycles. The animals were then randomly assigned to the healthy control group, placebo group, acupotomy group, and drug group, with 10 animals in each group. KOA was induced in all rabbits except those in the healthy control group. Two rabbits in each group were pathologically examined to confirm successful model establishment, and the remaining eight rabbits in each group were measured for other physiological indices. Animals were handled in compliance with the “Guideline on Humane Treatment of Laboratory Animals” by the Ministry of Science and Technology of China in 2006 in all experiments.

### 2.2. Reagents and Instruments

Bandage for plaster fixation (15 cm × 180 cm, Livzon Medical Biological Materials Co., Ltd.), needle knife (0.4 mm × 40 mm, Jiangsu Hu ayou Medical Devices Co., Ltd.), diclofenac diethylamine emulgel (Beijing Novartis Pharma Co., Ltd., lot no.: VP1735), primers (Sangon Biotech), TRIzol (Life Technologies), SDS kit (Sigma), hematoxylin (BA-4041) and eosin staining solutions (BA-4022) (Baso Diagnostic Inc., Zhuhai, China), TUNEL assay kit (green) (Beyotime), DAPI staining solution (Beyotime), antifluorescence quenching mounting solution (Beyotime), RIPA cell lysis buffer (Beyotime), SDS-PAGE gel preparation kit (Beyotime), ECL hypersensitive luminescence kit (Thermo Fisher Scientific, USA), PVDF membrane (Beyotime), and primary and secondary antibody washing solutions for Western blot (Beyotime) were used. The following antibodies were used: (1) *β*-actin: TA-09, Zs-BIO; (2) PI3K p85: bsm-33219 M, Bioss; (3) PI3K p110: BS6052, Bioworld; (4) AKT2: bs-2056R, Bioss; (5) AKT3: bs-5146R, Bioss.

Olympus BX51 light microscope (Olympus, Japan), Yaguang YB-7LF paraffin dispenser (Telewell Electronics Limited), Leica RM2016 microtome (Leica, Germany), JEM1230 transmission electron microscope (JEOL, Japan), BA410E fluorescence microscope (Motic), high-speed desktop refrigerated centrifuge (Anhui Jiawen Instrument Equipment Co., Ltd.), LX 300 microcentrifuge (Kylin-Bell Lab Instruments Co., Ltd.), quantitative PCR instrument (Thermo Fisher Scientific, USA), EPS 300 electrophoresis apparatus (Tanon), VE-180 electrophoresis cell (Tanon), VE-186 semidry transfer cell (Tanon), and ImageJ software were used.

### 2.3. Model Construction

KOA was induced in the placebo group, acupotomy group, and drug group by fixing the knee in the extended position with plaster for 6 consecutive weeks according to Videman's methods [14]. Briefly, the left hind limb of rabbits was fixed in an extended position (180°) and wrapped in plaster (previously softened in 65–85°C water) from the groin to the ankle. Before the plaster hardened completely, a soft iron wire was looped around the leg to fix the plaster and the toes were exposed for the observation of blood supply. The plaster bandage should be removed immediately in case of apparent toe swelling, and the knee joints should be fixed again after the swelling subsides. Loose or unraveled plaster bandage should be tightened immediately.

**Figure 1:**
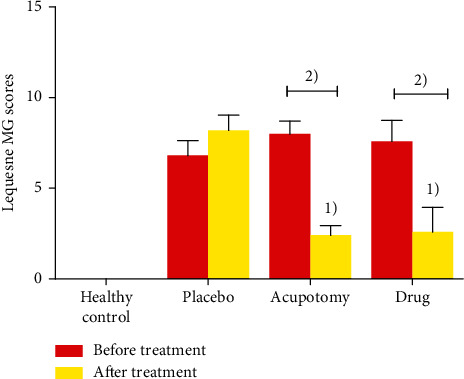
Lequesne MG knee joint evaluation of knee joint activity in each group. ^1)^*P* < 0.05 compared with the placebo group after treatment; ^2)^*P* < 0.05 compared with the same group after treatment.

The activity of animals in each group was evaluated after model establishment and treatment according to the modified Lequesne MG knee joint evaluation methods [15] (Figure 1). Two animals were randomly selected from each group for pathological examination of articular chondrocyte morphology.

### 2.4. Interventions

The healthy control group was fed a normal diet without any intervention. The placebo group was fed a normal diet after model establishment without any intervention. For the acupotomy group, treatment began 1 week after a successful model establishment. Briefly, the midpoints of the patellar ligament and medial/lateral collateral ligaments of the left knee joints were first marked by a surgical gentian violet marker as the needle knife insertion sites. Acupotomy was then performed following routine skin preparation and disinfection. (1) Releasing the patellar ligament: the skin was incised using a needle knife parallel to the patellar ligament. Once the patellar ligament was exposed, the blade was rotated 90° to make 3–5 incisions across the ligament. The knife was removed with the incision pressed using sterile gauze for hemostasis. (2) Releasing the medial/lateral collateral ligaments: the skin was incised using a needle knife parallel to the medial collateral ligament. Once the medial collateral ligament was exposed, the blade was rotated 90° to make 3–5 incisions across the ligament. The knife was removed and the incision was pressed with sterile gauze for hemostasis. Similar procedures were performed on the lateral collateral ligament. Acupotomy, once a week, was performed for three consecutive weeks. For the drug group, diclofenac diethylamine emulgel was applied topically to the knee twice daily for 3 consecutive weeks starting one week after successful model establishment.

### 2.5. Measurement of Physiological Indices

#### 2.5.1. HE Staining of Chondrocytes

Rabbits were anesthetized by intraperitoneal sodium pentobarbital (0.3%, 30 mg/kg) and fixed on the operating table. The left knee joint was incised using a scalpel (size number 23), and the patellar ligament was severed to fully expose the cartilage between the tibial plateau and femoral condyle. Fresh cartilage tissues (1 cm × 1 cm) were excised from the medial and lateral femoral condyles, fixed in 10% formalin solution, stained by HE, sectioned, and examined under a light microscope. Three to five tissue sections were selected from each group, and articular chondrocyte morphology was examined from 2 to 3 fields of each section.

#### 2.5.2. Examination of Chondrocytes by Transmission Electron Microscope (TEM)

Several pieces of fresh cartilage tissues (3 mm × 3 mm × 3 mm) were excised from the medial and lateral femoral condyles using the methods described above and immersed in fixative for electron microscopy. Tissue samples were rinsed, fixed in osmic acid, dehydrated, embedded, and cut into ultrathin (60 nm) sections (4–6 sections per group) according to routine sample preparation procedures for TEM. Sections were stained with lead citrate and uranium acetate, and the morphology of chondrocytes in 3–5 randomly selected fields of each section was examined under a JEM1230 TEM and photographed.

#### 2.5.3. Detection of Apoptotic Chondrocytes by TUNEL Staining (Green Fluorescence)

Cartilages were excised from the medial/lateral femoral condyles and tibial plateau of the left knee joint as previously described. Fresh cartilage tissues were fixed in 10% formalin solution. After fixation, the specimens were dehydrated, embedded in paraffin, and continuously sectioned at the coronal plane for at 5 *μ*m. The sections were washed thrice with 0.01 M phosphate buffer solution (PBS). Tissue sections were incubated in a 50 *μ*L TUNEL test solution for 60 min at 37°C. 6-Diamidino-2-phenylindole (DAPI) was used to tag the nucleus after washing thrice with PBS. Tissue sections were quenched and mounted for examination under a fluorescence microscope. Blue fluorescence represents normal cells and green fluorescence represents apoptotic cells. Three fields with fluorescence staining were photographed from each section.

#### 2.5.4. Real-Time Quantitative PCR (qRT-PCR)

Cartilages were excised from the medial/lateral femoral condyles and tibial plateau of the left knee joint as described above and stored at −80°C for subsequent analysis. PI3K and Akt expression in rabbit knee cartilage was measured using qRT-PCR. Briefly, tissue samples (50–100 mg each) were cut into pieces and homogenized in liquid nitrogen. Total RNA was extracted from the tissues using TRIzol, and RNA purity and concentration were measured using a spectrophotometer. Total RNA (3 *μ*g) was reverse transcribed into cDNA with a reverse transcription kit. PCR amplification was performed in a 10 *μ*L reaction containing 1 *μ*L cDNA template, 1 *μ*L each of forward and reverse primers, 5 *μ*L 2 × SYBR Green mixture, and 2 *μ*L RNase-free water. CT values of PI3K and Akt were obtained, and their relative expression was calculated by 2^−ΔΔCT^. Primer sequences and PCR product lengths are shown in Table 1.

#### 2.5.5. Western Blot

Cartilages were excised from the medial/lateral femoral condyles and tibial plateau of the left knee joint as described above and stored at −80°C for subsequent analysis. The protein expression of PI3K and Akt in rabbit knee cartilage was measured by Western blot. Briefly, tissue samples (approximately 100 mg each) were cut and homogenized in liquid nitrogen, lysed with 1 mL RIPA cell lysis buffer, and centrifuged at 12,000 r/min for 10 min. The supernatant was collected, and protein concentration was determined using the BCA assay kit as per the kit instructions. Protein samples were loaded and separated by SDS-PAGE gel electrophoresis, transferred onto a PVDF membrane, and blocked with 5% skimmed milk solution while shaking slowly at room temperature for 2 h. The membrane was incubated with primary and secondary antibodies and visualized using the ECL luminescence kit. The gray values of protein bands were analyzed using ImageJ software, and *β*-actin was used as the internal reference protein for calibration. Relative protein expression was calculated by the ratio of the gray value of the target protein band/gray value of the internal reference.

### 2.6. Statistical Analysis

Data were statistically analyzed using SPSS 22.0 and expressed as mean ± standard deviation ($\bar{x}$ ± *s*). Normally distributed data were compared among the four groups using one-way analysis of variance (one-way ANOVA) and between every two groups using the least significant difference (LSD) test. Nonnormally distributed data were compared using the Kruskal-Wallis H rank-sum test. The significance level was set to *α* = 0.05, and *P* < 0.05 was considered statistically significant. Statistical graphs were generated by GraphPad Prism 7.0.

## 3. Results

### 3.1. Lequesne MG Evaluation of Knee Joint Activity in Each Group

No significant differences in knee joint scores were observed among the model, acupotomy, and drug groups before treatment (Figure 1). Lequesne MG scores were significantly lower after treatment in both the acupotomy group and drug group (*P* < 0.05). Posttreatment Lequesne MG scores were also significantly lower in the acupotomy group and drug group than those in the placebo group (*P* < 0.05), but not significantly different between the acupotomy group and drug group (*P* > 0.05).

### 3.2. Pathological Changes in Articular Chondrocytes

**Figure 2:**
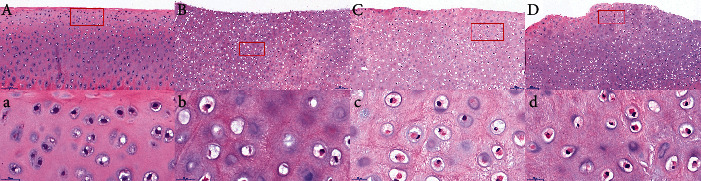
Pathological changes in knee joint chondrocytes in each group. A, a: healthy control group; B, b: placebo group; C, c: acupotomy group; D, d: drug group; a, b, c, d: enlarged views of the red boxes in A, B, C, and D, respectively. The scale is 100 *μ*m in A, B, C, and D and 20 *μ*m in a, b, c, and d.

Pathological examination showed that the knee joint of normal rabbits had a large number of neatly arranged chondrocytes, with many small, oblate, and separate immature chondrocytes near the perichondrium. In contrast, the placebo group had much fewer chondrocytes which were arranged in a disorganized fashion. Swelling, nucleus shrinkage and margination, and necrosis were also visible in some chondrocytes. Compared with the healthy control group, the placebo group displayed significant changes in the cartilage layer, including the presence of inflammatory cells in the cartilage layer and the absence of immature chondrocytes under the perichondrium. On the other hand, the acupotomy group and drug group had greater numbers of chondrocytes than the placebo group. These cells were neatly arranged and the cartilage layers were intact. Some immature chondrocytes were also visible near the perichondrium. The number of chondrocytes in the acupotomy group and drug group was basically the same, with little difference (Figure 2).

### 3.3. Comparison of the Microstructures of Knee Joint Chondrocytes

**Figure 3:**
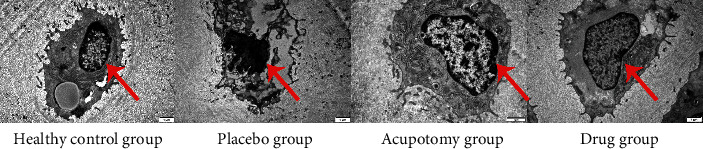
Comparison of the microstructures of knee joint chondrocytes (Scale = 1 *μ*m). The chondrocytes from the acupotomy group and drug group have slightly folded nuclear membranes with an intact membrane structure compared with the placebo group.

TEM showed evenly distributed chromatin in the nuclei with a clear and smooth nuclear envelope boundary in the healthy control group. The rough endoplasmic reticulum (ER) appeared normal in the cytoplasm of most chondrocytes but slightly swollen in a few cells, and the mitochondria were also intact. In contrast, in the placebo group, chondrocytes had shrunken nuclei and condensed chromatin that marginated to the nuclear envelope. Rough ER was nearly absent in the cytoplasm. There were also fewer mitochondria, some of which were swollen and degenerated. In the acupotomy group and drug group, nuclear chromatin condensation was reduced and margination was not apparent. The nuclear envelope boundary was relatively clear and smooth, and the rough ER appeared normal in the cytoplasm. Mitochondria with ruptured cristae were present in a few cells. There was no significant difference between the two groups (Figure 3).

### 3.4. TUNEL (Green Fluorescence) Staining of Knee Joint Chondrocytes

**Figure 4:**
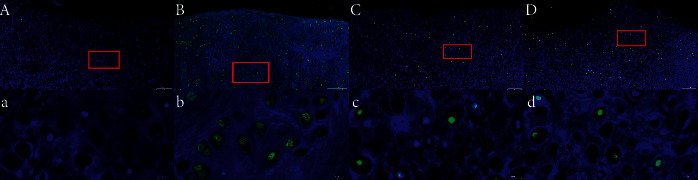
TUNEL (green fluorescence) staining of knee joint chondrocytes. A, a: healthy control group; B, b: placebo group; C, c: acupotomy group; D, d: drug group; a, b, c, d: enlarged views of the red boxes in A, B, C, and D, respectively. The scale is 100 *μ*m in A, B, C, and D and 20 *μ*m in a, b, c, and d.

**Figure 5:**
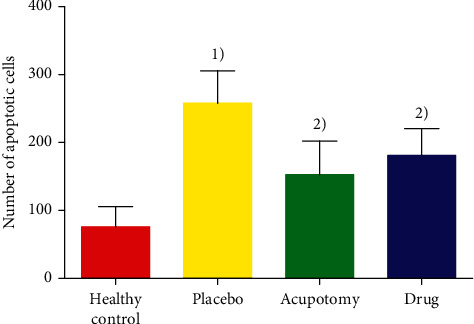
Comparison of the number of apoptotic chondrocytes. ^1)^*P* < 0.05 compared with the placebo group after treatment; ^2)^*P* < 0.05 compared with the same group after treatment.

Fluorescence microscopy demonstrated that the chondrocytes in the cartilage tissues were structurally distinct, with blue and green fluorescence indicating normal and apoptotic nuclei, respectively (Figure 4). The cartilage tissue of the healthy control group had oval chondrocytes that mostly fluoresced blue, indicating that there were more normal and less apoptotic chondrocytes. Green fluorescence was mainly located in the center of the cytoplasm of apoptotic cells. In contrast, the cartilage tissue of the placebo group showed an increased number of green fluorescent spots that were near the margin of the cells, indicating increased apoptotic chondrocytes. Nuclear chromatin condensation and margination were visible, and the cells were arranged in a disorganized manner. However, some cells were not fluorescent. The cartilage tissues of the acupotomy group and drug group had both blue and green fluorescence spots, but there were fewer apoptotic cells in these two groups than in the placebo group (*P* < 0.05). Although the acupotomy group had a fewer number of apoptotic cells than the drug group, the difference was not statistically significant (Figure 5).

### 3.5. Comparison of PI3K and Akt mRNA Expression in Knee Joint Cartilage

**Figure 6:**
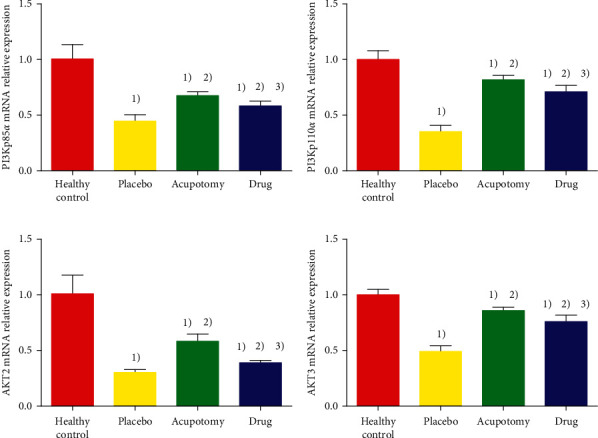
Comparison of PI3K and Akt mRNA expression in rabbit knee cartilage. (a) The ordinate represents PI3Kp85*α* mRNA relative expression. (b) The ordinate represents PI3Kp110*α* mRNA relative expression. (c) The ordinate represents AKT2 mRNA relative expression. (d) The ordinate represents AKT3 mRNA relative expression. ^2)^*P* < 0.05 compared with the healthy control group; ^2)^*P* < 0.05 compared with the placebo group; ^2)^*P* < 0.05 compared with the acupotomy group.

PI3K and Akt expression levels were significantly higher in the knee cartilage of normal rabbits than those in the placebo group, acupotomy group, and drug group (*P* < 0.05) and higher in the acupotomy group and drug group than those in the placebo group (*P* < 0.05). Compared with the drug group, the acupotomy group had significantly higher PI3K and Akt expression that was closer to the levels in the healthy control group (Figure 6).

### 3.6. Comparison of PI3K and Akt Protein Expression in Knee Joint Cartilage

**Figure 7:**
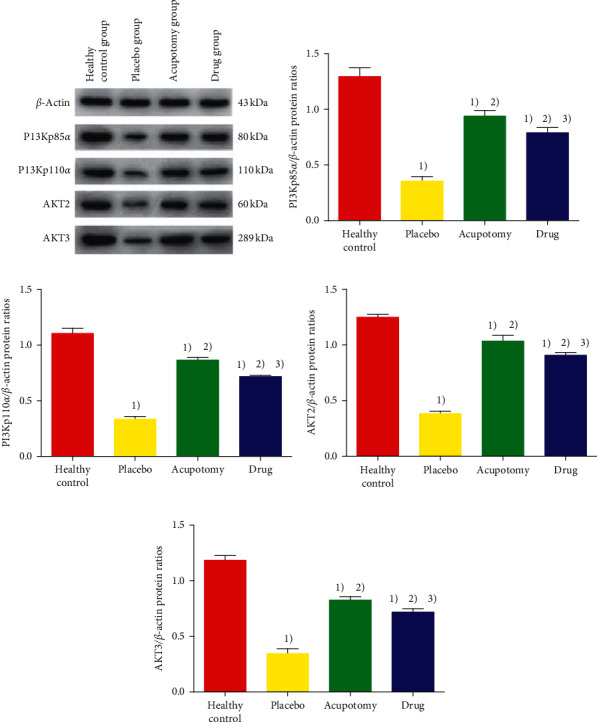
Comparison of PI3K and Akt protein expression in rabbit knee cartilage. (a) Western blot band diagram of rabbit cartilage PI3K and Akt protein in each group. (b) The ordinate represents PI3Kp85*α*/*β*-actin protein ratios. (c) The ordinate represents PI3Kp110*α*/*β*-actin protein ratios. (d) The ordinate represents AKT2/*β*-actin protein ratios. (e) The ordinate represents AKT3/*β*-actin protein ratios. ^2)^*P* < 0.05 compared with the healthy control group; ^2)^*P* < 0.05 compared with the placebo group; ^3)^*P* < 0.05 compared with the acupotomy group.

The protein expression of PI3K and Akt in knee joint cartilage was significantly higher in the healthy control group than that in the placebo group, acupotomy group, and drug group (*P* < 0.05) and higher in the acupotomy group and drug group than that in the placebo group (*P* < 0.05). In addition, PI3K and Akt protein expression was significantly higher in the acupotomy group than that in the drug group and was closer to the levels in the healthy control group (Figure 7).

### 3.7. Correlation between Chondrocyte Apoptosis and PI3K/Akt Protein Expression

**Figure 8:**
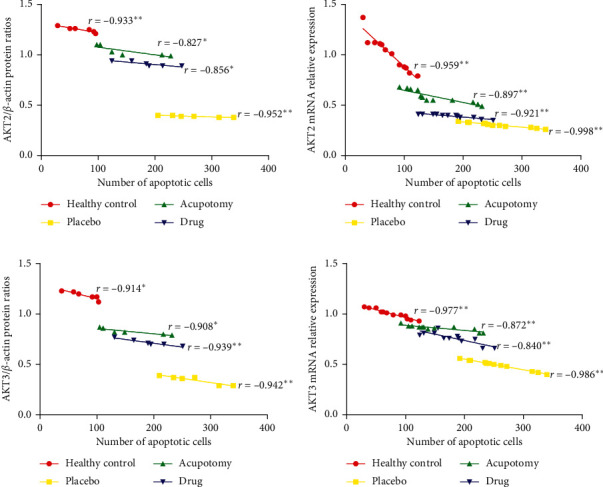
Correlation analysis of chondrocyte apoptosis with Akt expression in rabbit knee cartilage. (a, b) Correlation analysis between the expression of AKT2 at the protein and mRNA levels and the number of apoptotic cells. (c, d) Correlation analysis between the expression of AKT3 at the protein and mRNA levels and the number of apoptotic cells; *r* correlation coefficient. (a) The ordinate represents AKT2/*β*-actin protein ratios. (b) The ordinate represents AKT2 mRNA relative expression. (c) The ordinate represents AKT3/*β*-actin protein ratios. (d) The ordinate represents AKT3 mRNA relative expression. ^*∗*^*P* < 0.05, ^*∗∗*^*P* < 0.01.

In order to explore the possible mechanism of the P13K/Akt signaling pathway and chondrocyte apoptosis, we also analyzed the correlation between Akt protein and gene expression and the number of apoptotic cells. Pearson correlation analysis demonstrated that the correlation coefficients between rabbit knee joint chondrocytes AKT2 and AKT3 in protein and mRNA level expression and the number of apoptotic cells were less than 0 in each group. The absolute value of the correlation coefficient in the placebo group was higher than that in the healthy control group, acupotomy group, and drug group. The closer the absolute value of the correlation coefficient was to 1, the stronger the correlation was, and the difference was statistically significant (*P* < 0.05 or *P* < 0.01). PI3K/AKT signaling was negatively correlated with chondrocyte apoptosis in the knee joint cartilage in all groups (Figure 8).

## 4. Discussion

Articular cartilage degeneration is the key event in OA pathogenesis [16]. Chondrocyte apoptosis is one of the main factors leading to cartilage degeneration and can directly lead to the destruction of the articular cartilage, which affects the function of the joints, thus leading to OA development and progression [17]. Therefore, inhibition of chondrocyte apoptosis may facilitate the repair of damaged cartilage and thus alleviate the symptoms and control the progression of OA. Acupotomy can alleviate stress stimulation by incising the adhesions, contractures, and scars of joints. Acupotomy can ameliorate the stress inside and outside the joint and reduce the undermining cartilage pressure exerted on the joint [18, 19]. The metabolism of chondrocytes and the joint mechanical balance are thus improved, and the stability of the microenvironment within the joint is regulated. Consequently, chondrocyte apoptosis is inhibited and joint cartilage degeneration is alleviated.

In this study, we observed significant changes in the knee joint cartilage of KOA rabbits, including a few degenerated and necrotic chondrocytes and infiltration of inflammatory cells. The TUNEL assay showed significant chondrocyte apoptosis, chromatin condensation and margination, and disorganized cell arrangement, indicating the presence of inflammatory response and cartilage damage in the knee joint of KOA rabbits. In contrast, KOA rabbits that received acupotomy and drug intervention showed an increased number of neatly arranged chondrocytes, relatively intact cartilage layer, few immature chondrocytes near the perichondrium, and significantly reduced the number of apoptotic chondrocytes in the knee joint, which suggest that acupotomy and drug interventions inhibited chondrocytes apoptosis and repaired and reduced cartilage damage in the knee joints of KOA rabbits.

The PI3K/Akt signaling pathway, which is composed of PI3K, an intracellular phosphatidylinositol kinase, and Akt, an important downstream target in the PI3K signaling pathway, is involved in the regulation of cell apoptosis mainly mediated by Akt [20, 21]. PI3K/Akt signaling is ubiquitous in synovial cells during rheumatoid arthritis. The activated PI3K/AKT signaling pathway affects a variety of downstream effector molecules and leads to unbalanced apoptosis. Here, we found that PI3K and Akt mRNA and protein expression levels were downregulated in the chondrocytes of KOA rabbits, which indicates that the transmission of survival signals via the PI3K/Akt pathway was impaired in chondrocytes. This consequently leads to decreased chondrocyte growth, increased chondrocyte apoptosis, and accelerated chondrocyte senescence and degeneration.

Acupotomy is a new needle knife-based therapy that combines acupotomology, anatomy, and surgery. Acupotomy can promote soft tissue healing and restore the static balance of soft tissues by releasing soft tissues such as the muscles and fascia. This therapy has demonstrated satisfactory clinical efficacy in the treatment of KOA. We observed that KOA rabbits had upregulated PI3K and Akt mRNA and protein expression in the chondrocytes following acupotomy. The correlation coefficient between Akt expression and apoptosis increased significantly. We can increase the expression of Akt to promote the growth of chondrocytes while reducing the apoptosis of cartilage. Based on our findings and those from previous studies [22, 23], we speculate that acupotomy can activate the PI3K/Akt signaling pathway, thus regulating downstream target proteins to inhibit chondrocyte apoptosis and delay chondrocyte aging and degeneration. These effects help protect and improve the morphology of articular cartilage and are valuable for preventing and treating KOA.

Although KOA rabbits that received diclofenac diethylamine emulgel also showed upregulated mRNA and protein expression of PI3K and Akt, their levels were significantly lower compared with those in the acupotomy group. Acupotomy not only helps adjust the mechanical balance of the knee ligament by relieving soft tissue spasms near the knee joint but also mediates the biological changes of cartilage tissues to alleviate disturbances in the intra-articular microenvironment [18, 19, 24]. Therefore, acupotomy is superior to diclofenac diethylamine in repairing articular cartilage and delaying cartilage degeneration and joint damage. Chondrocyte apoptosis in the knee joint is associated with multiple signaling pathways that interact and influence each other [25–27].

In summary, our study showed that acupotomy suppresses chondrocyte apoptosis and promotes chondrocyte growth in KOA by upregulating the PI3K/Akt signaling pathway, which helps us understand how acupotomy takes effect in KOA. We can use this as a breakthrough in the treatment of KOA and achieve the goal of improving the quality of life of KOA patients. However, the mechanism of action of acupotomy in KOA is still very complex and warrants further investigation.

## Figures and Tables

**Table 1:** Primer sequences for RT-PCR of target genes.

Primer name	Amplicon length (bp)		Primer sequence
*β*-Actin	113	Forward	CAGCCCTCCTTCATCGGTAT
		Reverse	GACATGACGTTGTTGGCGTA

PI3Kp85*α*	182	Forward	AACCCTGCAGAACTACGACA
		Reverse	GGCGCTCTGTATTTCTTGGG

PI3Kp110*α*	182	Forward	GAGGTTTGGCCTGCTTTTGG
		Reverse	CTGGTTGCCTCATTTGCTCA

AKT2	132	Forward	ACGCATTCCAGACCCATGAC
		Reverse	AGACGATTTCCGCACCGTAG

AKT3	183	Forward	GAGGACCGCACACGTTTCTA
		Reverse	TGTCTTCATGGTGGCTGCAT

## Data Availability

The data used to support the findings of this study are available from the corresponding author upon request.
